# Beyond the “urge to move”: objective measures for the study of agency in the post-Libet era

**DOI:** 10.3389/fnhum.2014.00450

**Published:** 2014-06-20

**Authors:** Noham Wolpe, James B. Rowe

**Affiliations:** ^1^Department of Clinical Neurosciences, University of CambridgeCambridge, UK; ^2^Medical Research Council, Cognition and Brain Sciences UnitCambridge, UK; ^3^Behavioural and Clinical Neuroscience Institute, University of CambridgeCambridge, UK

**Keywords:** agency, voluntary action, Libet, objective measures, intentional binding, motor control, active inference, neuroimaging

## Abstract

The investigation of human volition is a longstanding endeavor from both philosophers and researchers. Yet because of the major challenges associated with capturing voluntary movements in an ecologically relevant state in the research environment, it is only in recent years that human agency has grown as a field of cognitive neuroscience. In particular, the seminal work of Libet et al. (1983) paved the way for a neuroscientific approach to agency. Over the past decade, new objective paradigms have been developed to study agency, drawing upon emerging concepts from cognitive and computational neuroscience. These include the chronometric approach of Libet’s study which is embedded in the “intentional binding” paradigm, optimal motor control theory and most recent insights from active inference theory. Here we review these principal methods and their application to the study of agency in health and the insights gained from their application to neurological and psychiatric disorders. We show that the neuropsychological paradigms that are based upon these new approaches have key advantages over traditional experimental designs. We propose that these advantages, coupled with advances in neuroimaging, create a powerful set of tools for understanding human agency and its neurobiological basis.

## Introduction

For centuries, the topic of human volition has been the playground and battlefield for philosophers and religious thinkers to debate the existence of “free will”, its role in driving human behavior, and its incompatibility with determinism. However, alongside its conceptual importance in the philosophical discourse, impairments in volition have also prompted the scientific investigation of the psychological processes and neurobiology of the sense of agency.

The sense of agency refers to the conscious experience that one has volitional or willed control over one’s own actions, and through these actions one can influence the environment. Agency is hence one component of the experience of awareness of actions, which includes, among other qualia, the sense of ownership over one’s body parts (Synofzik et al., 2008c). Agency research has attracted investigators and theorists for many years, but it is only in recent decades that human agency has become an active field of neuroscientific research (Haggard, 2008). This is partly due to the major challenges associated with capturing voluntary movements in an ecologically relevant state while in a research environment. Based upon this research, several theories have been developed to explain the origins of the sense of agency.

One prominent theory emphasizes the importance of predictive signals to agency (Blakemore et al., 2002). According to this “comparator” model, the sense of agency arises as a result of a comparison between predictive signals generated during motor planning and the actual sensory effect of one’s action. An action is perceived as self-caused in the case where there is a match between the predicted and actual sensory effect. A second account describes the experience of agency as a postdictive or retrospective insertion to consciousness—that is, an “editing” of the conscious experience after the action has already occurred (Wegner and Wheatley, 1999). In this “apparent mental causation” theory, an action is self-attributed when it follows one’s intention; has no other plausible causes and is consistent with the perceived outcome. There can be an integration of these predictive and postdictive cues (Synofzik et al., 2013), possibly through an optimal “cue integration” process (Moore and Fletcher, 2012), in which more reliable cues are given a larger weight for determining if an action is one’s own. These theories will be discussed in this Review in the context of specific agency measures.

The development of neurobiological theories for the sense of agency is largely the result of a recent boost in agency research. The seminal work of Libet et al. (1983) has substantially contributed to this growth, as it paved the way towards establishing a neuroscientific approach to studying human agency. Libet’s study differed from the early investigations of agency that were dominated by the use of *explicit* reports of intentionality and control by participants. For example, such experimental paradigms involve asking subjects to rate how much they felt in control of a certain movement, or whether a sensory stimulus was felt to be the result of their own action (Wegner and Wheatley, 1999; Wegner, 2003). As we discuss below, the application of such tasks is problematic, especially in the clinical population (e.g., Franck et al., 2001). The indirect and quantitative approach of Libet’s study has thus inspired the development of novel agency measures.

Over the last decade, new paradigms which draw upon emerging concepts from cognitive and computational neuroscience have been developed to investigate awareness and control of voluntary action without depending on subjective reports. Here we review the principal methods for examining agency with objective measures, including: (1) intentional binding which has its origins in the chronometric approach embedded in Libet’s study; (2) motor control theory and the comparator model; and (3) current and potential application of active inference theory.

We start off with Libet’s experiment as the key step triggering the development of indirect and quantitative measures for the neuroscience of agency, but also describe its caveats that have highlighted the need for objective measures. We then present the advantages that have made objective measures of agency so effective and review the three principal methods. Lastly, we show that when combined with advances in neuroimaging, these methods provide critical insights into agency in healthy individuals and in patients.

## Libet’s experiment and quantitative measures of agency

The study of Libet et al. (1983) was a landmark in the neuroscience of agency. The novelty of the experiment lies in the successful combination of an ingenious behavioral task with a neuroimaging technique (electroencephalogram) that provides neural markers of critical neurophysiological events in volitional actions. Libet’s pioneering experiment epitomizes the *chronometric* approach for agency.

To address the intricate questions surrounding voluntary actions, one can fractionate the process leading up to the execution of a movement. Voluntary action becomes a set of decision processes about, for example, what action to perform, when to perform it, or whether to perform it at all (Brass and Haggard, 2008). Libet’s experiment focused on the component of “when” in a voluntary action.

A generalized Libet task involves a self-paced movement, such as a button press, together with the use of a “clock” for estimating either the time of a movement or the time of being aware of the intention to move. In the original paradigm, subjects were asked to flex their right wrist or finger while attending a clock face made up of a revolving dot on a screen. There were three conditions in which subjects reported the clock position in three events: (i) when they felt an “urge to move” (called “W judgement”); (ii) when they moved (“M judgement”); and (iii) when they felt an unexpected skin stimulation (“S judgements”). Using electromyography to measure muscle activity, the judgements were compared against the veridical time of movement initiation. It was found that subjects perceive the time of their intention to move to occur about 200 ms prior to movement. Time of movement was perceived about 85 ms before movement onset, and time of sensory stimulation about 50 ms prior to stimulation.

Electrical brain activity was recorded by electroencephalography (EEG). The main aim was to compare subject judgement errors to the time of the readiness potential, the reliable negative potential measured by EEG before a voluntary movement (Kornhuber and Deecke, 1965). The conscious intention lagged the initiation of the readiness potential by about 300–500 ms. The finding indicates that brain activity in preparation for action starts before people are aware they want to perform an action, and therefore conscious awareness is unable to cause the brain activity for action execution.

Libet’s experiment kindled two main lines of research. First, many studies went on to investigate the behavioral and neural mechanisms of agency by examining the underlying mechanisms of the task, taking advantage of the quantitative nature of its measures. Second, because Libet’s paradigm measures the perceived times of events surrounding voluntary actions, the paradigm has provided indirect and arguably more objective measures of agency, compared to the self-reports of agency that have been traditionally used. These indirect and quantitative measures have been adopted for the study of patient populations, where reliable self-reports are often difficult to obtain. The emergence of an indirect approach for examining agency in Libet’s task was thus an important step towards the development of objective measures for agency.

The first line of research has examined the mechanisms of agency through Libet’s task. Although it remains debateable what exactly the W and M judgements reflect (Lau et al., 2006; Banks and Isham, 2009), neuroimaging studies have exploited the continuous and quantitative measures in order to link them with activity of specific brain regions. EEG data showed that the W judgement is more closely related to the lateralized component of the readiness potential, suggesting that it is linked to the time when a motor plan is specified (Haggard and Eimer, 1999). Functional MRI has been used to examine the roles of attention to intention and attention to action in the task (Lau et al., 2004). Relative to attention to the M judgement, attention to the W judgement is associated with increased activity in the pre-supplementary motor area (pre-SMA), dorsolateral prefrontal cortex (PFC) and intra parietal sulcus of the posterior parietal cortex (PPC; Lau et al., 2004). In contrast, the M judgement is associated with activity in the cingulate motor cortex in the mid-posterior aspect of the medial frontal cortex (Lau et al., 2006).

Striking evidence for an association between the W judgment and neural activity comes from Fried et al. (2011), using single cell neuron recording in humans. Neurons in the SMA, pre-SMA and anterior cingulate cortex predicted the time of W judgements. The authors proposed that an integration of these signals leads to conscious awareness of intentionality. Interestingly, an earlier study showed that stimulation of similar areas induces a similar experience of “urge to move” a specific body part (Fried et al., 1991). Taken together, these results reveal some of the complex neural substrate of agency.

The second line of research following up Libet’s paradigm has successfully used the task as a quantitative measure to study changes in awareness of action in clinical population (reviewed in Rowe and Wolpe, in press). For example, the sense of agency might be altered in Tourette’s syndrome by the repeated occurrence of involuntary movements or vocalizations known as tics, which are not perceived by patients as self-caused (Singer, 2005). This has motivated the investigation of agency in tic disorders and Tourette’s syndrome, demonstrating for example that the M judgement is unaffected in Tourette’s patients, whereas the W judgement is shifted positively towards the time of the movement. This change in W judgement is proportional to disease severity (Moretto et al., 2011).

The W judgement is also positively shifted towards the time of movement in patients with psychogenic movement disorders (PMD; Edwards et al., 2011). PMD is a constellation of movement disorders that result from a psychological or psychiatric disturbance, in which patients report the experience of motor symptoms without their control, although there is no organic neurological cause (Schrag et al., 2013). PMDs manifest a positive shift in the W judgement compared to controls, and also show a small shift in the M judgement, perceiving the time of movement as later than controls. The shift in W judgement is larger than that in M judgement, such that overall the two judgements do not differ in PMDs. The authors suggested the lack of temporal distinction between intention and action could explain how PMD patients perceive their psychogenic actions as involuntary, although these actions share similar neurophysiological correlates as healthy voluntary movements (Schrag et al., 2013).

Clinical studies suggest that Libet’s task can detect and quantify changes in the sense of agency. However, although Libet’s main results have been replicated in numerous studies (e.g., Matsuhashi and Hallett, 2008), studies using the paradigm have also raised methodological and interpretative limitations, which should be taken into account (e.g., see review of Roskies, 2010). One major criticism relates to the large individual differences in the use of the “clock” and potential biases in the time estimation procedure (Lau et al., 2006). This drawback hinders the interpretation of results from patient studies such as those presented above, which may simply represent different strategies to the task between patients and controls.

Another criticism surrounds the ambiguity in judging the time of an “urge to move”. As described above, the great advantage of Libet’s task was its indirect and somewhat more objective nature compared to direct judgements of agency, as it looks at the perceived times of events surrounding a voluntary action. However, particularly the W judgement requires an introspection of a conscious experience. Even if this conscious event of feeling an urge to move is real and discrete, the subjective account inherent in the Libet task retains the drawbacks of a direct approach, underscoring the need for fully objective measures.

In conclusion, Libet’s task has been subjected to the scrutiny of a multitude of replication studies and has given important insights by providing quantitative measures related to agency. However, due to its limitations and dependence on subjective experience of agency, there is a need for more objective measures of volitional actions, which we discuss in the next section.

## Beyond the “urge to move”: advantages of objective measures of agency

**Figure 1:**
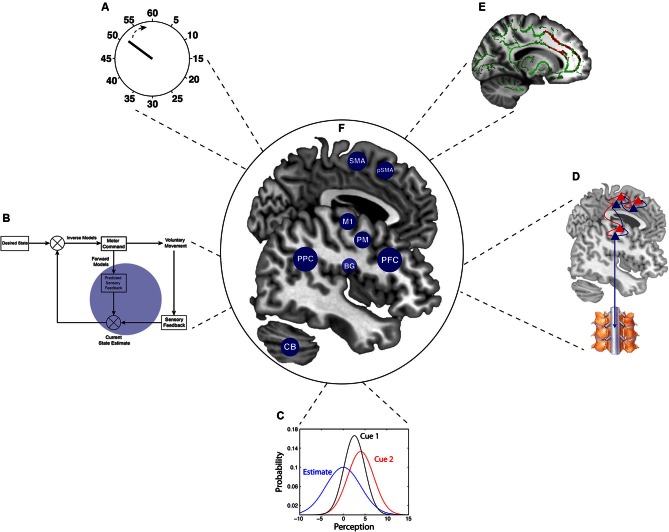
**Summary of the key theoretical concepts and neuroanatomy in the study of agency**. This review describes some of the key emerging neuroscientific concepts that have facilitated the acquisition of more objective measures in agency research. **(A)** The “clock” represents developments cognitive neuroscience paradigms, particularly the chronometric approach for volition that is embedded in Libet’s paradigm and in “*intentional binding”*. **(B)** *Comparator model* of agency within *optimal motor control* theory (enlarged in Figure 2). **(C)** Precision-dependent cue integration, following optimal Bayesian integration. **(D)** The recently developed theory of *active inference*, implementing Bayesian principles for voluntary action (enlarged in Figure 3). **(E)** Combination of structural and functional *neuroimaging* (inset adapted from Wolpe et al., 2014), which when considered together with these behavioral paradigms, provides a powerful tool for linking behavior to its underlying brain mechanisms. **(F)** The central brain illustration depicts the critical brain areas for voluntary action, which are alluded to in this Review. BG = basal ganglia; CB = Cerebellum; M1 = primary motor cortex; PFC = prefrontal cortex; PM = Premotor cortex; PPC = posterior parietal cortex; pSMA = pre-supplementary motor area; SMA = supplementary motor area.

Emerging concepts from cognitive and computational neuroscience (Figure 1) have led to novel experimental paradigms that indirectly map onto awareness and control of action through *objective* measures. Although the sense of agency is by definition a subjective conscious experience, it has been demonstrated that agency arises from the activity and interaction of different sensory and motor brain areas (Fried et al., 1991, 2011; Desmurget et al., 2009). An indirect approach exploits the integration of the sensory and motor systems in the central nervous system, and the effect of this integration in shaping and perceiving behavior (e.g., Hamilton et al., 2004; reviewed in Schütz-Bosbach and Prinz, 2007). Therefore, instead of metacognitive judgements of agency or time of intentions as in Libet’s task, these paradigms use low-level perceptual changes that are associated with volitional actions.

**Figure 2:**
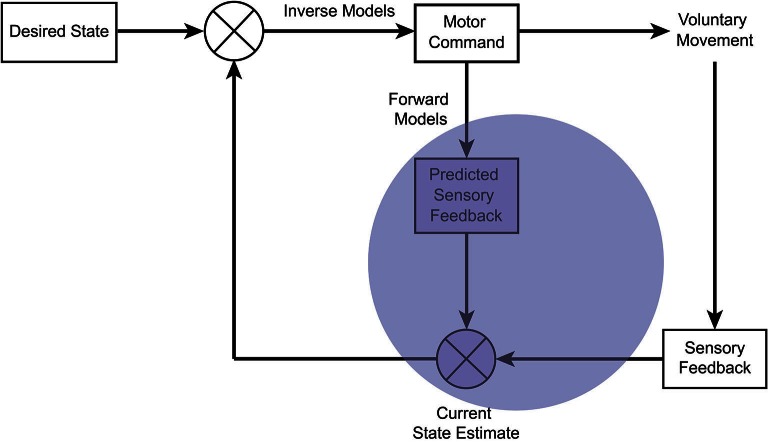
**Schematic of the “comparator model” within optimal motor control theory**. A prominent theory in motor control proposes the use of internal models, which represent the dynamics of the body in the environment. To generate a voluntary movement, the central nervous system represents a desired state of the body. This is compared with the estimated current state, and is converted to a motor command through inverse models by an optimal feedback controller, so as to minimize both the difference and the motor costs. An efference copy of the motor command is used by forward models to predict the sensory effect. The predicted sensory effect is compared and integrated with the actual sensory feedback from the moving body part to generate an optimal state estimate. According to the comparator model, the sense of agency arises from the comparison between the predicted and actual sensory feedback (opaque blue). When the discrepancy is small, the sensory effect is attributed to one’s own volition, but when the discrepancy is large, the sensory effect is interpreted as externally generated.

**Figure 3:**
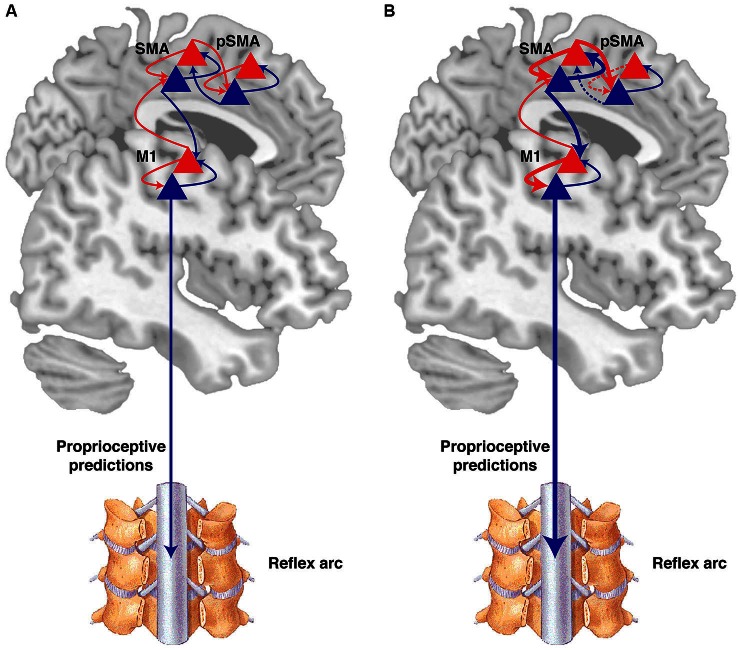
**Active inference and the sense of agency. (A)** According to the active inference theory, at each level of the cortical hierarchy, there are prediction units (blue triangles) representing the “belief” at each level, which modulate their activity so as to predict the “belief” or inference of the state at the level below. Backward projection (blue arrows), signal the belief to prediction error units (red triangles) at the same level and at the level below. The prediction error units project the error forward (red arrows). This hierarchical network converges on a minimized prediction error. Minimizing prediction errors can be achieved by adjusting the sensory information through movement. High level areas in a motor hierarchy, such as the pre-SMA (pSMA), signal beliefs or goal states as represented by their expected sensations to lower level areas, such as the SMA, which in turn project to the primary motor cortex (M1). Even lower level predictions are sent to the spinal cord, inducing movements through reflex arcs. The sense of agency arises from the consistency between predictions in high level and lower level sensory data, balancing precision across the network. **(B)** In psychogenic movement disorders (PMD) for example, there is a misallocation of attention, and intermediate-level areas gain abnormally high precision (thick arrows) (Edwards et al., 2012). Consequently, prediction errors at that level induce movements through lower levels of the hierarchy, and overwhelm the higher intentional levels that initially did not predict the movement (dashed arrows). This discrepancy makes the network converge on the most likely explanation that a movement was externally caused. The figure is based on Friston et al. (2012).

There are three principal advantages for such objective measures, particularly in patient populations. First, the implementation of quantitative and *objective* paradigms is not reliant on subjective reports and introspection, which might be biased or confounded. For example, there might be critical differences between the “feeling of agency” and “judgement of agency” (Synofzik et al., 2008b). A feeling of agency is the low-level perceptual experience of whether an action is self-caused, and it is proposed to be dependent on distinct processes in sensorimotor control (see next section). In contrast, judgement of agency is the explicit, high-order interpretation of being the agent of an action. The interpretation is dependent on the feeling of agency and additional signals, such as contextual information (Synofzik et al., 2008b). Moreover, the judgement of agency might indirectly influence the feeling of agency (Synofzik et al., 2013). Therefore, probing explicit agency reports through the judgement of agency might in fact introduce confounding factors, while biasing the measures of interest.

Second, in patient groups, metacognitive insights and self-monitoring can themselves be impaired, as seen in PMDs (de Lange et al., 2007; Pareés et al., 2012) and schizophrenia (Frith and Done, 1989). Such impairments are difficult to measure, but may interfere with direct measures of agency. For example, schizophrenia patients over-attribute sensory events to their own actions (Franck et al., 2001). However, these patients also tend to “jump into conclusions” based on less evidence, and ignore new evidence that supports an alternate inference (e.g., see review by Fletcher and Frith, 2009). The over-attribution of action might thus reflect abnormalities in decision making rather than in agency, and it is not straightforward to separate such metacognitive processes from the processes that are linked to agency.

Third, new objective paradigms can be designed to probe *specific* mechanisms within the volitional operation, in conjunction with the recent mechanistic insights into both normal and abnormal voluntary control. In patients, this advantage facilitates the achievement of two critical aims: (i) improving the understanding of clinical phenomenology by addressing more specific questions about the nature of disorders of agency and by providing candidate biomarkers for treatments; and (ii) using disorders as a model for testing mechanistic hypotheses regarding the neural substrates of agency. Meeting the latter aim could not only help mapping the functional anatomy for agency, but also test for causality (i.e., whether a brain area is causally involved in agency) and necessity (whether it is required for agency).

Together, these advantages have made objective measures appealing for the neuroscience of agency. We next review the main three advances in cognitive and computational neuroscience that have facilitated this research approach.

## Intentional binding: objective chronometry in the study of agency

The “intentional binding” paradigm evolved from Libet’s task: subjects use a “Libet clock” to report the time of either an action, such as pressing a button, or the time of a sensory event, such as a tone. When the action and the sensory event are coupled together, subjects tend to perceive their action as occurring later in time and the consequent sensory event as occurring earlier in time, relative to when both events occur separately. Importantly, this temporal attraction or the binding of an action and its sensory consequence does not occur for passive or involuntary TMS-induced actions (Haggard et al., 2002; Engbert et al., 2008), and is interestingly related to explicit judgement of control in some cases (Ebert and Wegner, 2010).

Intentional binding can be generalized to actions and sensory consequences of different modalities (Engbert et al., 2008), but most studies use an auditory tone. The principal measures include binding of action (the delay in the perception of action and its attraction towards the time of tone) and binding of tone (the earlier perception of tone and its attraction towards the time of action) (Haggard et al., 2002). One can also examine “composite” binding, in terms of the sum of action binding and tone binding (Moore et al., 2010b; Moore and Obhi, 2012), although there are caveats to this approach (see below).

Intentional binding measures have already proved advantageous in the study of agency (Moore and Obhi, 2012). The paradigm elegantly overcomes some of the innate limitations of Libet’s task. As binding is a relative measure, the paradigm successfully addresses many critical confounds of Libet’s task, mainly the individual differences in strategy or biases in time estimation procedure. Crucially, it does not require subjects to report conscious reflections, as in an urge to move, making it an objective chronometric measure for the study of agency.

There are, however, unresolved issues and limitations of the paradigm that should also be carefully considered. Temporal binding is not limited to one’s own actions, and can also occur when observing the actions of another agent (Wohlschläger et al., 2003) or even when observing a predicted action-effect sequence generated by a machine (Buehner, 2012). These findings suggest that intentional binding might simply reflect the temporal binding resulting from learning the causal relations between actions and their effects, and cast doubts on the specificity of binding to one’s own actions and sense of agency. Further, intentional binding is usually observed on a group level, but there is a large single-subject variability, and many individuals do not show the effect (e.g., Wolpe et al., 2013). The source of this high variability remains largely unknown. Lastly, the paradigm can be subjected to a similar criticism as Libet’s task with regard to the need for dividing attention between the action and the clock, as well as the tone event in the binding task. These concerns emphasize the importance of examining the underlying mechanisms of binding.

Since it was introduced, the mechanisms of intentional binding have been extensively investigated. The relative contribution of predictive and postdictive or retrospective processes to binding of action has been examined using a modified intentional binding task. Moore and Haggard (2008) included two operant conditions: one in which the action triggered a tone in 50% of the trials; and another in which the action triggered a tone in 75% of the trials. Baseline measures were subtracted from the operant conditions as in the typical binding task. Binding of action was stronger for higher tone probability, but still occurred in trials with lower tone probability when the action was followed by a tone. These results suggest that binding of action results from a combination of predictive and postdictive signals (Moore and Haggard, 2008). Predictive signals for binding of action could come from an “efference copy” of motor commands (see next section). In contrast, the postdictive contribution to binding of action could be mediated through a precision-dependent integration of predictive signals with the time of action itself and its sensory effect (Wolpe et al., 2013).

Interestingly, a similar precision-dependent integration of predictive and postdictive signals has also been suggested to govern the correct attribution of actions (Moore and Fletcher, 2012), both in terms of the “feeling” and “judgement” of agency (Synofzik et al., 2013). The argument is that with optimal cue integration one can make better estimates by combining different sources of information, for example about the most likely time or cause of an action. The relative contribution of each source of information depends on its reliability (i.e., whether it is variable or noisy). As in binding of action (Wolpe et al., 2013), the sense of agency itself can be the outcome of a combination of predictive cues related to motor planning processes and postdictive signals, driven from sensory and high-level contextual agency cues. The cues are integrated as a function of their reliability and availability in each particular situation (Synofzik et al., 2013). The combination of such cues is an intriguing link between the mechanisms of binding of action and the sense of agency.

In contrast to binding of action, binding of tone might be more directly associated with sensorimotor prediction (Waszak et al., 2012; Wolpe et al., 2013). According to this notion, preparatory motor processes normally lead to a pre-activation of the neural representation of the predicted sensory effect of one’s action. When the sensory effect occurs, it reaches the perceptual threshold faster due to the increased excitability of the appropriate sensory representation (Waszak et al., 2012), resulting in a shortening of the perceptual latency (Wolpe et al., 2013). The magnitude of this effect can be shaped by high-level beliefs about the cause of the action, which in turn does not influence binding of action (Desantis et al., 2011). These examples for a mechanistic discrepancy between action and tone binding suggest that these measures could be more informative when considered separately, which is illustrated next as we review studies of intentional binding.

Wolpe et al. (2014) have used the intentional binding paradigm in combination with multimodal brain imaging, to study the mechanisms of agency through the disorders of agency associated with the corticobasal syndrome (CBS; Wolpe et al., 2014). CBS is a progressive asymmetric movement disorder often caused by cortical and subcortical degeneration (Gibb et al., 1989). CBS is associated with two disorders of volitional actions: alien limb (the performance of semi-purposeful movements in the absence of “will”) and apraxia (in this case the impairment in the performance of complex movements despite the understanding of their goal). We used intentional binding to investigate possible abnormalities in agency in the more severely affected limb.

In CBS patients, tone binding was normal in both hands compared to controls. In contrast, there was a specific increase in binding of action in the more-affected hand. Binding was normal in the less-affected hand, providing a crucial internal control condition that rules out general task deficits. Moreover, the magnitude of action binding correlated with the severity of alien limb and apraxia. The substantial increase in action binding was interpreted through the lens of cue integration theory: a low reliability (or high uncertainty) in the perception of time of action could lead to an over-reliance on the sensory effect for the perception of one’s own action. Supporting this interpretation, the precision of time estimates in baseline conditions correlated with action binding, as predicted by the cue integration theory (Wolpe et al., 2013). The authors proposed that the volitional signals that drive internally generated actions (and suppress actions triggered by the environment) were imprecise due to gray and white matter degeneration.

Intentional binding is also abnormal in patients with PMDs (Kranick et al., 2013). In these patients, however, there was no difference in binding of action, but a consistent reduction in the binding of tone. As binding of tone is strongly reliant on intact predictive processes for agency, the results suggest a specific prediction abnormality in PMD which has been confirmed by complementary methods as illustrated in the next sections.

Abnormal binding is found in patients with schizophrenia in proportions to symptoms of delusions (Voss et al., 2010). Almost 80% of schizophrenia patients present with delusions or false beliefs, many of which implicate the sense of agency, such as delusions of control or passivity phenomena (Andreasen and Flaum, 1991). Voss et al. (2010) used the modified binding task from Moore and Haggard (2008) that is described above and quantified the relative contribution of predictive and postdictive signals. The predictive component was calculated by subtracting judgement errors in the “action only” trials (i.e., when actions were not followed by tones) in the 50% tone probability condition, from judgement errors in “action only” trials in the 75% tone probability condition. The retrospective component was calculated by subtracting judgement errors in the “action only” trials in the 50% tone probability condition from judgement errors in the “action and tone” trials (i.e., when actions were followed by tones) in the 50% tone probability condition. Patients showed a substantially diminished predictive contribution, but an increased retrospective contribution for the perception of time of action in binding of action. Interestingly, the reduction in the predictive component was related to severity of positive symptoms. The authors suggested that the abnormally high association between actions and effects in schizophrenia results from an over-reliance on retrospection, due to impaired prediction (Voss et al., 2010).

A recent development has been the characterization of pharmacological contributors to agency. For example, the NMDA antagonist ketamine enhances binding of action (Moore et al., 2011), while dopamine replacement therapy in Parkinson’s disease (PD) increases overall intentional binding (Moore et al., 2010b). These results suggest that dopamine (or its interactions with NMDA) can modulate the sense of agency. Further development of this pharmacological perspective is anticipated in the next few years, with major implications for treating disorders of agency.

Despite the potential limitations of the paradigm, intentional binding can objectively quantify essential aspects of agency in health and disease. The task can improve the understanding of agency when considered together with its underlying mechanisms of postdictive and predictive volitional processes, the importance of which is further emphasized in the next section.

## Optimal motor control theory and the comparator model of agency

The indirect investigation of agency and awareness of action has drawn on concepts from optimal motor control theory. The principles underlying this line of research are: (a) awareness of action arises from specific processes in motor control (the “comparator” model); and (b) experimental tools that probe motor control processes are applicable to the awareness of action (Frith et al., 2000; Blakemore et al., 2002). In this section we expand these principles, and illustrate how they have been implemented in clinical populations.

Optimal motor control theory draws on engineering principles and a general hypothesis of internal models (Figure 2): to optimize motor control, the central nervous system internally represents the dynamics of one’s own body and its interaction with the external world (Wolpert, 1997; Wolpert and Ghahramani, 2000). These models are learned and updated to reliably represent the relationship between motor commands and their sensory effects.

An inverse model generates the appropriate motor command for movement according to a comparison between the current state of the body and the goal. Optimization balances performance accuracy and the motor costs (Todorov and Jordan, 2002; Scott, 2004), while a forward model uses an “efference copy” of the motor command (von Holst, 1954) to predict the sensory effect of an action. The predicted sensory effect is integrated with the actual sensory feedback by precision-dependent Bayesian integration (see Figure 1C). The combination of prior knowledge (predictions of the forward model) with sensory evidence (actual sensory feedback) generates a “posterior” distribution for the state estimate (Wolpert et al., 1995), which in turn is used to update the motor command.

Within these processes, the comparator model suggests that the sense of agency arises from the comparison between the predicted and actual sensory feedback (Frith et al., 2000). If the predicted sensory effect matches the actual sensory effect, a sensation is perceived as self-caused. However, when there is a large discrepancy, a sensation is perceived as externally generated, independent of one’s own volition. In turn, deficits in any of the processes of the comparator may underlie abnormalities in the awareness and control of action (Frith et al., 2000; Blakemore et al., 2002). The comparator model within optimal motor control theory has thereby provided a useful framework for addressing questions surrounding the mechanisms that underlie agency in health and disease (Rowe and Wolpe, in press).

The comparator model, however, cannot explain some aspects of the experience of agency. For example, not all divergences from the predicted sensory effect reach awareness, and small sensory discrepancies or their ensuing motor adjustments do not necessarily influence the sense of agency (Castiello et al., 1991; Fourneret and Jeannerod, 1998). The model has also been criticized for not encompassing external contextual cues, such as the emotional valence of sensory effect or high level beliefs about an action (Synofzik et al., 2008b, 2013). The importance of such postdictive indicators of agency is emphasized in the “apparent mental causation” theory (Wegner and Wheatley, 1999; Wegner, 2003). These cues have been demonstrated to influence not only the explicit judgement of agency (Wegner, 2003), but also the lower experience of feeling of agency as measured by intentional binding (Moore et al., 2009; Desantis et al., 2011).

Nevertheless, there is currently little doubt as for the importance of action planning signals, particularly sensorimotor prediction, and the processing of sensory feedback for the sense of agency. The most recent theories of agency have thus argued for an integration between the sensorimotor signals embedded in the comparator model and the high-level postdictive cues for generating a sense of agency (Moore and Fletcher, 2012; Synofzik et al., 2013).

In what follows, we review the current research looking at the role of the sensorimotor signals rooted in the comparator model for impairments of agency. We first consider studies that have pointed to an abnormal sensorimotor prediction, followed by studies of abnormal processing of sensory feedback and their implications for the sense of agency in patients.

### Sensorimotor prediction

Intact sensorimotor prediction is typically linked to the fundamental difference between the perception of self-generated and externally triggered sensory stimuli. For example, the inability to tickle oneself is dependent on accurate spatio-temporal predictions (Blakemore et al., 1999). Such difference between the perception of self- and externally triggered sensations is captured by “sensorimotor attenuation”, i.e., the reduction in the perceived intensity of the consequences of one’s own actions relative to externally caused sensations (Shergill et al., 2003). The attenuation is temporally centered on the time of the action, and relies on accurate sensorimotor prediction (Bays et al., 2005, 2006).

Two main explanations for attenuation have been put forward. One suggests that it is directly linked to the efference copy that is used by an internal model to generate a predicted sensory intensity. The predicted sensory intensity is in turn removed from the actual sensory feedback for the perception of the consequences of one’s action (Bays et al., 2006). A more recent account posits that attenuation results from a predictive activation of the sensory representations of the prospective sensation. This “pre-activation” reduces the sensitivity to the actual sensory stimulus (Roussel et al., 2013). In either case, attenuation has a critical behavioral role, facilitating the distinction between the effects of self-generated actions and external sensory events. Normal sense of agency thus relies on intact prediction and its consequent sensorimotor attenuation, which may in turn provide a measure for the integrity of agency.

A robust method to measure sensorimotor attenuation is a “force matching” task. In the original task of the haptic modality, varying forces were applied to subjects’ left index finger by a lever attached to a torque motor. Subjects were asked to reproduce the forces by pressing the lever with their right index finger (Shergill et al., 2003). Typically, the reproduced forces are larger than the forces that are actually applied by the torque motor. The degree of overcompensation has been used as a proxy for sensorimotor attenuation and the integrity of agency.

In PMDs, the extent of overcompensation is reduced compared to controls, such that patients show a more “accurate” perception of the sensory consequences of their actions, similar to external sensory events (Pareés et al., in press). These prediction deficits in PMD may thus lead to the abnormal perception that their movements are involuntary and not self-caused (Schrag et al., 2013), as suggested by the results from Libet’s task.

Reduced attenuation is also reported in schizophrenia (Shergill et al., 2005), and has been linked to delusions of agency. This association is further supported by the correlation between visual sensory attenuation and the severity of delusions (Lindner et al., 2005). Thus, increasing impairments in sensorimotor prediction in schizophrenia and the inability to “remove” self-caused sensory information for perception are tightly linked to delusions of influence and abnormalities in agency.

Sensorimotor prediction was elegantly probed by Lindner et al. (e.g., Synofzik et al., 2006), drawing on the methodology of classic motor adaptation paradigm. Subjects performed out-and-back ballistic pointing movements, and receive visual feedback through a mirrored computer screen, while the true position of their hand was not visible. A deviation in the visual feedback was introduced, and subjects learned to correct for this perturbation. Two additional components were added to this conventional motor adaptation task: a perceptual component, wherein subjects indicate the perceived position of their action outcome; and a motor test component, wherein subjects point to a target in the absence of feedback (Synofzik et al., 2006).

Subjects normally learn to correct their movement in the presence of an initial deviation in the visual feedback. Interestingly, after learning to correct for the deviation, subjects perceive their movement as deviant even when no visual feedback is given, and move accordingly when asked to point to a target, suggesting they internally update their predictions. Predictions are thus adaptable, enabling the correct attribution of new sensory outcomes to one’s own action (Synofzik et al., 2006). The authors used the task to test awareness of action in patients with cerebellar lesions (Synofzik et al., 2008a) and in schizophrenia (Synofzik et al., 2010).

Cerebellar patients of mixed pathologies showed intact discrimination thresholds for detecting feedback perturbation in the sensory effect of their movement. Patients also adapted their movement similarly to controls when visual feedback was given throughout the movement (Synofzik et al., 2008a). However, when no online feedback was given, the cerebellar group showed reduced perceptual adaptation than controls. Patients also compensated less for the experienced deviation when asked to point to a target. These results suggest that awareness of action in cerebellar patients could remain intact, but might be affected when predictions require adjustments, e.g., when the dynamics with the environment change (Synofzik et al., 2008a).

In contrast, schizophrenia patients demonstrated increased thresholds for detecting feedback perturbation in movements. The magnitude of the increase positively correlated with the severity of delusions of influence (Synofzik et al., 2010). Moreover, schizophrenia increased adaptation to the deviated feedback when it was displayed, but when no feedback was given their updated perception and adjusted movements were similar to controls (Synofzik et al., 2010). The results corroborate force matching and intentional binding data, highlighting an over-reliance on sensory feedback for the perception of actions in schizophrenia.

### Processing of sensory feedback

According to the comparator model, impaired agency could also arise from impairments in sensory processing (see Figure 2). Changes in sensory processing in relation to awareness of action has been investigated in the context of kinaesthetic deficits in PD. PD is associated with neuronal dysfunction and loss in the substantia nigra, which can result in muscular rigidity, resting tremor, bradykinesia and slowness in the initiation of voluntary movements (Hughes et al., 1992). PD also affects a wide range of sensory and cognitive functions, including the perception of one’s own movement.

Kinaesthesia (the awareness of the position and movement of one’s body parts) is impaired by PD. For example, patients require larger passive limb displacements for becoming aware of such displacement (Konczak et al., 2007). By optimal motor control theory, kinaesthesia might rely on efferent signals from sensorimotor prediction, as well as afferent signals from the moving body part, such as proprioceptive and haptic information. The origins of kinaesthetic abnormalities was investigated by Konczak et al. (2012).

An age-related decline in haptic perception was found, with a strong trend towards an increase in detection thresholds, but stable discrimination thresholds. In PD, both detection thresholds and discrimination thresholds were increased (Konczak et al., 2012). The thresholds were similarly increased both when patients actively explored a virtual contour surface and when their hand was passively moved on the surface. As both conditions require intact processing of sensory feedback, this shared deficit is likely to arise mainly from impaired low-level processing of afferent signals. Abnormal afferent signals could thus contribute to abnormal kinaesthesia and awareness of movement and position of one’s body limb in PD (Konczak et al., 2012).

To sum up, a growing number of studies employ concepts from optimal motor control theory in the comparator model to investigate agency. In addition to their objective nature, the additional value of these studies lies in their capacity to reveal specific mechanisms that are required for normal sense of agency and its changes in patient populations. We next review an alternative theory to optimal motor control for voluntary action, and its current and potential applications for the study of agency.

## Active inference: a new approach to the understanding of agency

The previous section underscored the importance of sensorimotor prediction for voluntary control and for the sense of agency. It also emphasized the role of prediction deficits in disorders of agency, e.g., in PMDs and in schizophrenia. Prediction in the brain can also be framed in terms of the “free energy” principle, according to which the brain constantly seeks to minimize its “surprise” (Friston, 2010). Surprise in this context amounts to unexpected sensations or “prediction errors”, including those that are contingent on one’s own action (Friston, 2010). This principle can explain several perceptual phenomena and in recent years has been extended to encompass voluntary actions (Friston, 2011) and disorders of agency (e.g., Edwards et al., 2012) under a unifying “active inference” theory.

In order to explain how prediction errors give rise to voluntary action and agency, it helps to first consider the origins of this theory in predictive coding for perception. Helmholtz proposed that perception is a process of probabilistic inference, whereby the brain infers the sensory causes based on certain sensory effects (von Helmholtz, 1860). Combined with the free energy principle, it has been proposed that perceptual inference relies on hierarchical predictive processing (Friston, 2010; Clark, 2013). Accordingly, higher levels in a cortical hierarchy adjust their predictions so as to “explain away” sensory samples from the lower levels (Figure 3A).

Specifically, at each level of the cortical hierarchy there is a set of neurons encoding predictions, and another set encoding prediction errors (“prediction units” and “prediction error units”). Prediction units encode the “belief” at that level, i.e., the probabilistic representation of the causes of sensation, and provide prediction signals through top-down (backward) projections to prediction error units at the level below (Feldman and Friston, 2010; Friston, 2010; Clark, 2013). Prediction error units receive prediction signals from the level above and compare them to the sensory belief at that level. The discrepancy constitutes the prediction error, which is projected forward to the higher cortical level that adjusts its predictions, so as to minimize the prediction error it receives (Feldman and Friston, 2010; Friston, 2010; Clark, 2013). The process of minimizing prediction errors by adjusting predictions at each level of the hierarchy allows different levels of representation of the causes of the sensory input—and that is perception.

Hierarchical predictive processing is implicitly Bayesian in that the sensory representation or belief at each hierarchical level is analogous to the Bayesian posterior distribution. It is derived from a precision-dependent combination of both prior beliefs (prediction signals) and likelihood or sensory evidence (prediction error signals) (Friston, 2010). The precision of the prediction error at each level is important for determining the balance between prior beliefs and sensory evidence for perception. The relative precision of prediction errors is suggested to be determined as a function of post-synaptic gain, modulated by neuromodulators, and optimized through attention (Feldman and Friston, 2010).

Predictive coding can be extended to explain voluntary action in the “active inference” theory (Friston et al., 2010). In the sensory system, perception is proposed to result from minimization of prediction errors in different levels of the cortical hierarchy through the adjustment of predictions (or beliefs). In the motor system, minimizing prediction errors is achieved by adjusting the sensory data through movement (Figure 3A). Expectations of the sensory consequence thus drive the movement of limbs through classical motor reflex arcs, so as to “fulfil” the prediction signals. In other words, movement is specified in terms of the expected sensation (Friston et al., 2010). This theory for voluntary action has been applied to explain movement disorders and abnormalities in the sense of agency in patients.

PMD has been suggested to result from a misallocation of attention (Edwards et al., 2012) with abnormally high precision of prior beliefs at intermediate levels of the cortical hierarchy (Figure 3B). As a result, the abnormally precise intermediate priors are spread down the hierarchy to the spinal cord where they induce abnormal movements through the reflex arcs. In parallel, the abnormally precise prediction errors are propagated forward to higher “intentional” levels in the hierarchy (i.e., levels where activity is more directly related to conscious awareness of action), such as the pre-SMA. As the relative precision of representations at the higher levels is reduced, prediction errors at the intermediate levels overwhelm the high-level intentional priors, and indicate a movement that was not predicted by the higher levels. The discrepancy between high intentional levels that do not predict movements and the abnormally precise intermediate levels leading to movements, causes the abnormal movements to be interpreted as involuntary, without one’s sense of agency (Edwards et al., 2012).

In psychosis, abnormal awareness of action has been proposed to result from a perturbed inference as a result of aberrant encoding of precision (Adams et al., 2013). Here, abnormal release of neuromodulators, such as dopamine, together with altered post-synaptic NMDA receptor densities in PFC, lead to reduced precision of high-level prior predictions. These may lead to false perceptual inferences and catatonia. For example, the suppression of high-level predictions result in their inability to induce movements, and consequently in akinesia (Adams et al., 2013). The catatonic state could be rescued by a compensatory increase in the precision of probabilistic representations in intermediate levels. In this case, low-level proprioceptive data does not predominate, allowing top-down prediction from the intermediate levels to induce movements. However, the compensatory increase in intermediate precision now leads to a mismatch between intentional and lower levels of the hierarchy as in PMD, making the patient prone to a misattribution of action and abnormal agency (Adams et al., 2013).

On these active inference accounts, the sense of agency arises from the capacity of higher intentional levels of the cortical hierarchy (e.g., pre-SMA, PFC) to predict sensory data from lower levels (SMA, M1) through movement. Critically, normal agency depends on a balance in the precision of prediction errors within the cortical hierarchy for action, and the ability of this balanced hierarchy to converge on the most likely cause of a sensation. The theory thus offers a different and novel research avenue for the objective investigation of agency, focusing on testing parameters of brain connectivity within hierarchical networks.

A similar approach has been successfully implemented to investigate the sensory system. For example, the hypothesized modulation of the precision of prediction errors by the neuromodulator acetylcholine has been supported in a multimodal study (Moran et al., 2013) incorporating the mismatch negativity paradigm (Näätänen et al., 1978), dynamic causal modeling (Friston et al., 2003; Rowe et al., 2010a) and a pharmacological manipulation. Moreover, it has been shown that individual differences in connectivity in a hierarchical sensory network can not only underlie behavioral changes in a perceptual task, but also relate to delusional ideation of healthy participants (Schmack et al., 2013).

Although the active inference theory has not yet been applied experimentally for the study of agency, this approach can already be implemented in the research lab. Experiments of active inference on agency could include a behavioral task involving a voluntary action, such as a simple action selection task (Rowe et al., 2010b), which triggers activity in the key areas for action (as in Figure 1F). One could then use dynamic causal modeling to reveal variability in connectivity measures within hierarchical networks for agency, resulting from either individual differences, pharmacological manipulation or disease state. Moreover, new sensorimotor paradigms that probe different levels of prediction for voluntary action will be able to shed light on their underlying neural mechanisms and on the differential contribution of distinct levels of prediction to the sense of agency.

Active inference provides an appealing attempt to develop mechanistic accounts for the sense of agency, among diverse cognitive and motor phenomena. Importantly, it offers a unified account by integrating psychophysical and clinical observations with structural and functional brain imaging. The advantages of combining neuroimaging with new agency studies are discussed in the final section.

## An aperture to agency: combining objective measures with neuroimaging techniques to unravel the mechanisms of agency

As highlighted in the previous section, human brain imaging enables one to study the widely distributed networks related to agency. However, early neuroimaging studies of agency focused on contrasting self vs. externally triggered movements and contrasting different levels of perturbations to the sensory feedback. These univariate analyses implicated several areas, including the insular cortex, premotor cortex, cerebellum and the SMA and pre-SMA of the medial frontal cortex (Deiber et al., 1999; Farrer and Frith, 2002; Wiese et al., 2004; Rowe et al., 2010a, 2008; Rowe and Siebner, 2012).

As more advanced neuroimaging techniques have evolved, and in combination with computational modeling methods, neuroimaging studies have begun to point at more specific mechanisms of agency. Multivariate pattern analysis enabled the decoding of intentions from the frontopolar cortex several seconds before they reached awareness (Soon et al., 2008). Other methods include the application of accumulation-to-threshold models for predicting neuronal or BOLD signal in relation to voluntary actions (Zhang et al., 2012). This approach has shown that based on an increase in the firing rate of single neurons in the medial frontal cortex, it is possible to predict the time of awareness of the urge to move in Libet’s task (Fried et al., 2011). Such data suggest that the sense of agency emerges when activity of neurons in high-level areas, such as the pre-SMA reaches a certain threshold.

The advances in neuroimaging methods can be combined with lesions or clinical disorders. For example, Wolpe et al. (2014) studied patients with alien limb and apraxia resulting from the neurodegenerative CBS (Wolpe et al., 2014). They combined multimodal brain imaging with two of the three main advances discussed throughout this Review, namely: (i) the quantitative and objective measure of agency of intentional binding and (ii) a mechanistic account of agency that draws on optimal motor control theory. They showed how such a combination leads to a clear and integrated model of agency and its abnormality.

Patients with CBS showed a specific increase in binding of action measure of intentional binding in their more-affected hand, relative to their less-affected hand and to controls. The extent of the increase correlated with severity of alien limb and apraxia, suggesting that abnormally enhanced binding of action reflected the abnormalities in agency in CBS (Wolpe et al., 2014). Structural neuroimaging of voxel-based morphometry and diffusion tensor imaging showed that the gray matter volume in the pre-SMA and the white matter tract integrity of its connections, were associated with the specific behavioral change in action binding. Finally, functional connectivity at rest between the pre-SMA and PFC was increased as a function of enhanced action binding. Drawing upon the contribution of a precision-weighted integration to binding of action (Wolpe et al., 2013), the results suggest that there is reduced precision in the volitional signals that drive movements in CBS patients. The reduced precision was associated with impairments in a medial frontal-prefrontal network for agency and volitional control, with its hub in the pre-SMA (Wolpe et al., 2014).

Intentional binding was also combined with temporary “lesions” in healthy adults by transcranial magnetic stimulation. Stimulation over the pre-SMA, reduced intentional binding of the outcome tone (Moore et al., 2010a). As binding of the outcome tone is mainly driven by a reduction of perceptual latencies through sensorimotor prediction (Waszak et al., 2012; Wolpe et al., 2013), these results further suggest that the pre-SMA contributes to the sense of agency through the processing of specific predictions of the sensory effect.

We propose that the combination of advanced neuroimaging techniques with recent developments in the study of agency, and particularly the objective measures of agency, provide a powerful tool for an integrated study of agency. This approach can be applied to clinical and pharmacological investigations, thereby improving treatments for disorders of agency.

## Conclusions

We have reviewed the development and use of objective measures in the study of agency. We began by showing how Libet’s experiment was central to the development of the neuroscience of agency by providing indirect quantitative measures, and by inspiring the development of objective measures. These indirect objective measures are based on the chronometric approach in intentional binding, the comparator model of optimal motor control and the emerging active inference theory. We have discussed the advantages of objective measures especially in combination with advanced structural and functional neuroimaging techniques. We propose that this combination of methods and their application to patient populations will be important in the ongoing endeavor to discover the mechanisms of human agency.

## Conflict of interest statement

The authors declare that the research was conducted in the absence of any commercial or financial relationships that could be construed as a potential conflict of interest.
